# Metformin treatment in late middle age improves cognitive function with alleviation of microglial activation and enhancement of autophagy in the hippocampus

**DOI:** 10.1111/acel.13277

**Published:** 2021-01-14

**Authors:** Maheedhar Kodali, Sahithi Attaluri, Leelavathi N. Madhu, Bing Shuai, Raghavendra Upadhya, Jenny Jaimes Gonzalez, Xiaolan Rao, Ashok K. Shetty

**Affiliations:** ^1^ Institute for Regenerative Medicine Department of Molecular and Cellular Medicine Texas A&M University College of Medicine College Station TX USA

**Keywords:** activated microglia, cognitive function, metformin, neurogenesis, neuroinflammation

## Abstract

Metformin, a drug widely used for treating diabetes, can prolong the lifespan in several species. Metformin also has the promise to slow down age‐related cognitive impairment. However, metformin's therapeutic use as an anti‐aging drug is yet to be accepted because of conflicting animal and human studies results. We examined the effects of metformin treatment in late middle age on cognitive function in old age. Eighteen‐month‐old male C57BL6/J mice received metformin or no treatment for 10 weeks. A series of behavioral tests revealed improved cognitive function in animals that received metformin. Such findings were evident from a better ability for pattern separation, object location, and recognition memory function. Quantification of microglia revealed that metformin treatment reduced the incidence of pathological microglial clusters with alternative activation of microglia into an M2 phenotype, displaying highly ramified processes in the hippocampus. Metformin treatment also seemed to reduce astrocyte hypertrophy. Additional analysis demonstrated that metformin treatment in late middle age increased adenosine monophosphate‐activated protein kinase activation, reduced proinflammatory cytokine levels, and the mammalian target of rapamycin signaling, and enhanced autophagy in the hippocampus. However, metformin treatment did not alter neurogenesis or synapses in the hippocampus, implying that improved cognitive function with metformin did not involve enhanced neurogenesis or neosynaptogenesis. The results provide new evidence that metformin treatment commencing in late middle age has promise for improving cognitive function in old age. Modulation of microglia, proinflammatory cytokines, and autophagy appear to be the mechanisms by which metformin facilitated functional benefits in the aged brain.

## Graphical abstract

Ten weeks of metformin treatment in late middle age is efficacious for improving cognitive function in the old age. Improved cognitive function following metformin treatment was linked with antiinflammatory effects, the activation of adenosine monophosphate‐activated protein kinase (AMPK), the mammalian target of rapamycin (mTOR) signaling, and increased autophagy in the aged hippocampus. The results suggest that metformin therapy in middle age leads to better cognitive function in old age.

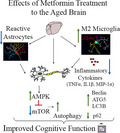

## 1 INTRODUCTION

Metformin (MET), a drug broadly used for treating type 2 diabetes mellitus (T2DM), has caught considerable attention as a promising anti‐aging drug because of its ability to prolong the lifespan in several species (Chaudhari et al., 2020; Rotermund et al., 2018). MET treatment in early or late middle age also appears attractive because both animal and human studies have implied that dysregulation of insulin function contributes to aging and age‐related neurodegenerative diseases (Craft & Watson, 2004; Verdile et al., 2015). It is believed that MET treatment slows down the process of aging through better insulin signaling, which postpones the age‐related neurodegenerative diseases. However, MET is yet to be accepted as an anti‐aging drug due to diverging animal and human studies results.

Clinical studies have mostly implied that MET has positive effects on cognitive function. For instance, MET use in patients with T2DM was found to be associated with a lower risk of cognitive impairment (Ng et al., 2014). Furthermore, the incidence of dementia seemed to be reduced in T2DM patients receiving MET (Hsu et al., 2011). Besides, the risk of developing Alzheimer's disease (AD) appeared to be lower in people with diabetes receiving MET than in patients receiving other diabetes medications (Cheng et al., 2014; Orkaby et al., 2017). A few studies have also suggested that long‐term use of MET for T2DM is associated with a higher risk of developing AD or cognitive impairment, however (Imfeld et al., 2012; Moore et al., 2013). Another study showed that 8 weeks of MET treatment improved executive function but not in other cognitive tests in nondiabetic patients with mild cognitive impairment (MCI) or mild AD‐related dementia (Koenig et al., 2017). Thus, MET’s effect on individuals’ cognitive function seemed to diverge depending on the risk profile of the patient receiving the drug and on complex underlying pathological processes (Wang et al., 2017).

The effects of MET on cognitive function in preclinical studies are also controversial. In the high‐fat diet studies, MET reduced cognitive deficits in three investigations, but another study found no improvement (Allard et al., 2016; Lennox et al., 2014; McNeilly et al., 2012; Pintana et al., 2012). Furthermore, two studies using db/db mice, an animal prototype displaying spontaneous mutation with insulin resistance and obesity, reported opposite results, one reporting no beneficial effects (Li et al., 2012) and the other showing improved memory function (Chen et al., 2016). Furthermore, MET alleviated cognitive impairments in an animal model treated with scopolamine (Mostafa et al., 2016) or subjected to hypobaric hypoxia (Zhao et al., 2019). About the effects of MET on aging, a study has reported that 8 weeks of MET treatment through drinking water (2 mg/ml) in 22‐month‐old male C57BL6/J mice impaired the spatial memory retrieval function with no changes in spontaneous motor activity or spatial learning (Thangthaeng et al., 2017). However, it was unclear whether the administration through drinking water resulted in a MET concentration sufficient to activate the energy regulator adenosine monophosphate‐activated protein kinase (AMPK) by phosphorylation in the aged brain. This issue is significant because AMPK activation is one of the primary mechanisms by which MET is believed to produce beneficial effects (Rotermund et al., 2018).

Thus, the effects of MET in aged animals on cognitive function are yet to be ascertained through MET treatment regimens that are efficient to enhance the phosphorylation of AMPK. Hence, in this investigation, we quantified the efficacy of 10 weeks of MET treatment (100 mg/kg via oral gavage) to improve cognitive function in 18‐month‐old male C57BL6/J mice. We specifically tested whether long‐term MET treatment commencing in late middle age would maintain better cognitive function in old age. Our results demonstrated improved pattern separation, object location, and recognition memory function in mice receiving MET compared to age‐matched mice receiving no treatment. Improved brain function was associated with reduced incidence of microglial clusters, increased density of M2 microglia with no effects on neurogenesis or synaptic proteins in the hippocampus. Moreover, MET treatment diminished the concentration of proinflammatory cytokines, activated AMPK, modulated the mammalian target of rapamycin (mTOR) signaling, and increased autophagy in the hippocampus.

## 2 MATERIALS AND METHODS

### 2.1 Animals and study design

Male C57BL6/J mice (n = 59) purchased from Jackson Laboratories were employed. Of these, 48 were in late middle age (18‐month‐old mice), and 11 were young adults (~3‐ to 4‐month‐old mice). The current study focused only on male mice due to sex differences in behavioral pattern separation ability, neurogenesis, synaptic plasticity, and transcription during the estrus cycle (Foster, 2005; Yagi et al., 2016). All animals, housed in an environmentally controlled room with a 12:12‐h light: dark cycle, received food and water *ad libitum*. The experimental procedures conducted were approved by the Animal Care and Use Committee of Texas A&M University.

The experiments were performed following two weeks of acclimatization to the vivarium. First, to discern cognitive function status in late middle age, young adult and 18‐month‐old mice (*n* = 11–12/group) were interrogated with a series of neurobehavioral tests. Next, for studying the effects of long‐term MET treatment on cognitive function, two groups of mice were randomly assigned either to the control aged or aged‐MET groups (*n* = 12/group, total, 24 mice). Animals in the aged‐MET group received MET via oral gavage (100 mg/kg/day in 0.5 ml distilled water; once daily, 5 days/week; Sigma‐Aldrich) for 10 weeks. To neutralize the potential confounds associated with handling and oral gavage, we gave mock oral gavage (without administering the drug) to mice in the control aged group. The weight of animals was comparable between aged and aged‐MET groups at the commencement of the study (aged, mean ± SEM, 38.7 ± 1.3 g; aged‐MET, 42.3 ± 3.5 g), and at different time‐points after the commencement of treatment (2 weeks, 38.7 ± 1.0 g versus 41 ± 3.6 g; 6 weeks, 38.8 ± 1 g versus 41.2 ± 3.7 g; 9 weeks, 38.5 ± 1 g versus 39.2 ± 3.8 g). The behavioral tests commenced in the 8th week of the treatment period and completed in the 10th week of the treatment period. The animals in control aged group were untreated, but behavioral tests were done in parallel to the aged‐MET group. The animals were euthanized for histological analysis immediately after the completion of behavioral tests. The animals in both groups were 21 months old at the time of tissue harvesting. Furthermore, to understand the molecular mechanisms underlying MET‐mediated beneficial effects, another cohort of mice (*n* = 6/group, total, 12 mice) received MET (100 mg/kg/day, once daily) or no treatment for 2 weeks. The animals were euthanized 3 h after the last treatment, and the blood samples were collected from the heart for measuring blood glucose concentration. The brain tissues were harvested, snap‐frozen, and stored at −80° centigrade for various biochemical analyses. The investigators who performed the behavioral tests and biochemical analysis were blinded to group identification of animals or samples. The animal numbers for behavioral studies, and histological and biochemical studies were determined through power analysis using G*Power software.

### 2.2 Pattern separation test

The pattern separation test (PST) employed in this study encompassed four consecutive trials (T1–T4), with each lasting 5 min and separated by an inter‐trial interval (ITI) of 30 min. See the supplemental file for details.

### 2.3 Object location test

The object location test (OLT) also included three trials (T1–T3), with each trial lasting 5 min and trials separated by an ITI of 15 min. See the supplemental file for details.

### 2.4 Novel object recognition test

The test comprised three trials (T1–T3), with each lasting 5 min and separated by an ITI of 15 min. See the supplemental file for details.

### 2.5 Tissue processing and immunohistochemistry, and quantification of microglia, astrocytes, and DCX+ newly born neurons

The animals were deeply anesthetized, perfused with 4% paraformaldehyde, the brain tissues were post‐fixed, and processed for cryostat sectioning following cryoprotection. Thirty‐micrometer thick coronal sections through the forebrain were collected serially, and every 15th section through the entire septotemporal axis of the hippocampus was processed for immunohistochemistry. See the supplemental file for details.

### 2.6 Morphometric analysis of IBA‐1+ microglia and GFAP+ astrocytes

The overall morphology of IBA‐1+ microglia and GFAP+ astrocytes from aged and aged‐MET groups was measured by tracing the soma and processes using a semi‐automatic cell tracing system (Neurolucida, Microbrightfield) linked to a Nikon microscope. See the supplemental file for details.

### 2.7 Immunofluorescence staining and confocal microscopic analyses

Immunofluorescence studies comprised detecting pAMPK expression among 4′,6‐diamidino‐2‐phenylindole (DAPI) neurons, and alternatively activated CD206+ M2 type microglia among IBA‐1+ microglia in the cerebral cortex and the hippocampus of aged and aged‐MET groups (*n* = 4–5/group). Additional immunofluorescence studies comprised visualization of Syn+ presynaptic puncta and PSD95+ postsynaptic puncta, and p62+ structures using tissue sections through the hippocampus from the young adult, aged, and aged‐MET groups (*n* = 5/group). See the supplemental file for details.

### 2.8 Measurement of Syn+, PSD95+, and p62+ structures

For measuring changes in Syn+ and PSD95+ puncta density and putative synapses (i.e., junctions of Syn+ and PSD95+ puncta) in the stratum radiatum of CA1 and CA3 subfields, we employed 0.5**‐**micrometer thick Z‐sectioning of tissue samples from young adult, aged, and aged‐MET groups. Our blinded analyses comprised quantification of: (a) the area fraction of Syn+ and PSD95+ puncta in randomly chosen 303 µm^2^ images (*n* = 5/subfield/group) using ImageJ; (b) the mean number of Syn+ and PSD95+ puncta in randomly selected 4 µm^2^ areas (10 areas/image, *n* = 5/subfield/group); and (c) the cumulative number of putative synapses (i.e., junctions of Syn+ and PSD95+ puncta) in randomly selected 4 µm^2^ areas (10 areas/image, *n* = 5/subfield/group). The area fraction of p62+ structures in the CA3 subfield was also quantified in a blinded manner using Image J (2 images/animal/group).

### 2.9 Biochemical assays using hippocampal lysates and the serum

We measured the effect of 2 weeks of MET treatment on the concentration of proinflammatory markers TNF‐α, IL‐1β, and MIP‐1α, mitochondrial complex I, oxidative stress markers MDA and protein carbonyls, pAMPKα, mTOR complex, and autophagy proteins beclin‐1, ATG5, and MAP1‐LC3B in the hippocampus of 18‐month‐old mice. See the supplemental file for details.

### 2.10 Statistical Analysis

Two‐tailed, unpaired, Student's *t* test in the Prism software was used to compare two columns of data within groups in behavioral tests or the data between aged and aged‐MET groups. The Mann–Whitney U test was employed when standard deviations between groups were found to be statistically significant. One‐way ANOVA with Student–Newman–Keuls post hoc tests was employed for comparisons involving three groups. The values mentioned in the bar charts are mean ±SEM, and *p* < 0.05 was considered statistically significant.

## 3 RESULTS

We first determined the extent of cognitive dysfunction in late middle age by interrogating young adult (*n* = 11) and 18‐month‐old (*n* = 12) male C57BL/6J mice with a series of neurobehavioral tests. The tests evaluated the ability of animals for pattern separation, perceiving minor changes in the environment (a measure of location memory), and novel object recognition memory. Following this, two groups of 18‐month‐old male mice were randomly assigned either to the control aged or aged‐MET groups (*n* = 12/group) to investigate the effects of long‐term MET treatment for improving cognitive function. We administered MET orally (via oral gavage) at a dose of 100 mg/kg (once daily, 5 days/week) and the treatment continued for 10 weeks. In the last 3 weeks of MET treatment, animals were evaluated for pattern separation ability and location and recognition memories.

### 3.1 MET treatment in late middle age improved pattern separation function

**Figure 1:**
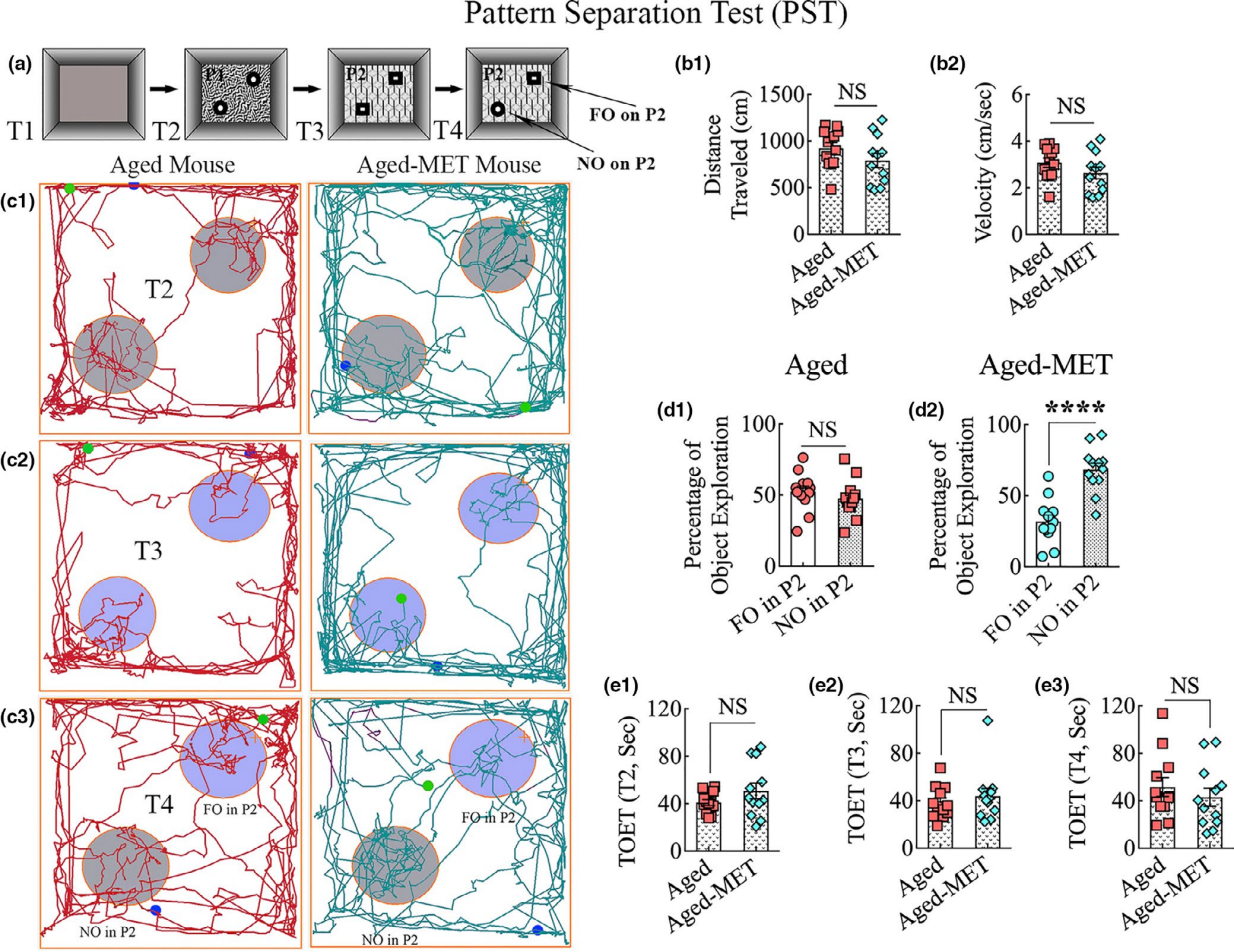
Ten weeks of MET treatment in the late middle age improved ability for pattern separation in old age. The cartoon in a shows the various trials (T1–T4), objects, and floor patterns involved in a pattern separation test. The bar charts in b1 and b2 compare distances traveled (b1) and the velocities of movement (b2) in T1, showing that motor function was comparable between the aged and aged‐MET groups. c1–c3 are tracings illustrating the movement in an open field and the exploration of object areas by an aged mouse (left panels) and an aged‐MET mouse (right panels) in T2–T4. The bar charts in d1 and d2 compare the percentage of exploration of familiar and novel objects on pattern 2 (FO or NO on P2) in T4 by aged mice (d1) and aged‐MET mice (d2). Note that aged‐MET mice displayed a preference for exploring NO on P2 in comparison to aged mice showing no such preference. The bar charts in e1‐e3 compare total object exploration times (TOETs) in T2–T4. ****p < 0.0001; NS, not significant

Pattern separation function reflects proficiency for discriminating similar but not identical experiences via the storage of similar representations in a non‐overlapping manner (Leutgeb et al., 2007). Pattern separation function depends on the integrity of DG‐CA3 circuitry in the hippocampus (Gandy et al., 2017). A PST was performed as described in our previous reports (Long et al., 2017). Briefly, the PST comprised four successive trials (trials 1–4, T1‐T4) with an inter‐trial interval (ITI) of 30 min (Figure 1 (a)). The trials comprised the acclimatization of the animal to the open field apparatus in T1 and the exploration of different objects on specific floor patterns in T2–T4. These include a pair of identical objects placed in distant areas on floor pattern 1 in T2, another pair of identical objects placed in distant areas on floor pattern 2 in T3, and one object from T2 and one object from T3 placed on floor pattern 2 in T4 (Figure 1 (a)). The object from T2 became a novel object on pattern 2 (NO on P2), whereas the object retained from T3 was a familiar object on P2 (FO on P2).

Young adult mice showed a proficiency for pattern separation, which was apparent from their increased propensity to explore the NO on P2 over the FO on P2 in T4 (*p* < 0.01; Figure S1 (b)). In contrast, 18‐month‐old mice exhibited pattern separation dysfunction, as they explored both NO and FO on P2 for nearly comparable periods (*p* > 0.05; Figure S1 (c)). Also, the total object exploration times (TOETs) were higher in young adults than 18‐month‐old mice (*p* < 0.05–0.001; Figure S1 (d–f)) in T2–T4, implying that the overall motor activity is reduced in late middle age compared to the young adult age.

In untreated aged mice (aged) and aged mice receiving MET (aged‐MET), the distances traveled and the velocities of movement in T1 were comparable (*p* > 0.05, Figure 1 (b1, b2)), suggesting that MET treatment did not alter the motor function of aged mice. The mice in both groups explored objects for significant durations in T2–T4. Representative tracings depicting the movement and exploration of objects by an aged mouse and an aged‐MET mouse in T2–T4 are illustrated in Figure 1 (c1–c3). Untreated aged mice displayed persistence of pattern separation impairment, which was apparent from their behavior, exhibiting no preference for the NO on P2 over the FO on P2 in T4. They spent nearly similar amounts of their object exploration time with the NO and the FO (*p* > 0.05, Figure 1 (c3, d1)). In contrast, aged‐MET mice displayed the ability for pattern separation, which was evident from their exploration of the NO on P2 for longer durations than the FO on P2 in T4 (*p* < 0.0001, Figure 1 (c3, d2)). The TOETs did not differ significantly between the two groups (Figure 1 (e1‐e3)) in T2‐T4. The distances traveled, or velocities of the movement, were also comparable between aged and aged‐MET groups in all trials (data not illustrated). These data confirmed that the outcomes were not influenced by varying object exploration times or motor deficits in one or the other group. Thus, the hippocampus‐dependent pattern separation function could be improved in aged mice through long‐term oral administration of MET.

### 3.2 Aged mice receiving MET displayed improved object location memory function

**Figure 2:**
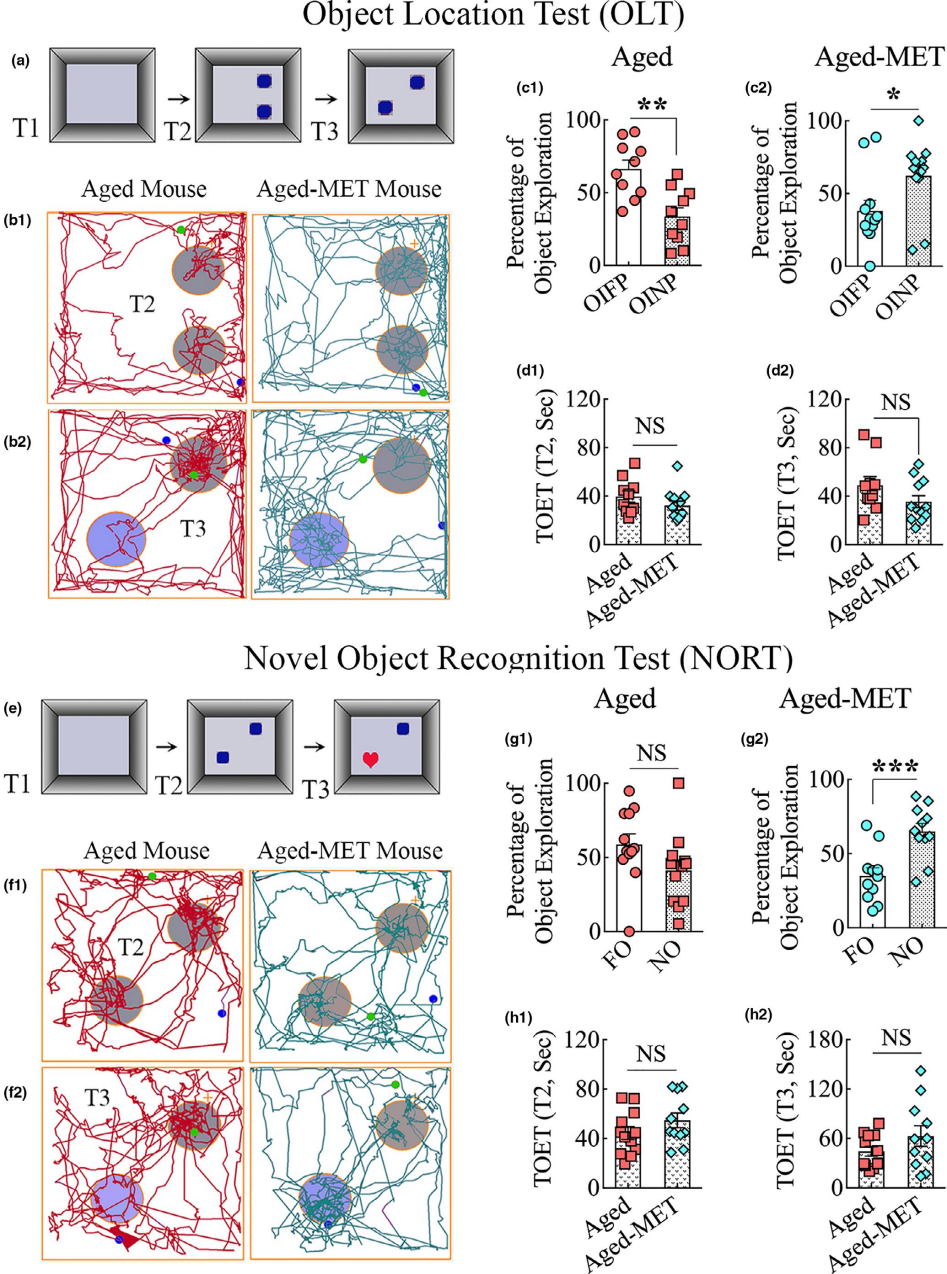
Ten weeks of MET treatment in late middle age improved object location and recognition memory function in old age. Cartoon a illustrates the trials (T1–T3) and objects employed in an object location test. b1–b2 are tracings depicting the movement in an open field and the exploration of object areas by an aged mouse (left panels) and an aged‐MET mouse (right panels) in T2–T3. The bar charts in C1 and C2 compare the percentage of exploration of the object in a familiar place (OIFP) vis‐à‐vis the object in a novel place (OINP) in T3 by aged mice (c1) and aged‐MET mice (c2). Note that aged‐MET mice showed a higher tendency for exploring the OINP than the OIFP. In contrast, the untreated aged mice showed greater affinity for exploring the OIFP over the OINP. The bar charts in d1 and d2 compare total object exploration times (TOETs) in T2‐T3. *p < 0.05; **p < 0.01; NS, not significant. The cartoon in e shows the various trials and objects employed in a novel object recognition test. f1–f2 are trials depicting the movement in an open field and the exploration of object areas by an aged mouse (left panels) and an aged‐MET mouse (right panels) in T2–T3. The bar charts in g1 and g2 compare the percentage of exploration of familiar and novel objects (FO or NO) in T3 by aged mice (g1) and aged‐MET mice (g2). Note that aged‐MET mice preferred to explore the NO over FO (g2), whereas the untreated aged mice showed no such preference (g1). The bar charts in h1 and h2 compare TOETs in T2–T3. ***p < 0.001

An OLT, a hippocampus‐dependent cognitive test, was utilized to examine the ability of mice to discern minor changes in the environment. Briefly, the test comprised three trials, each of which lasted 5 min, and the ITI was 30 minutes (Figure 2 (a)). The animals explored two identical objects placed on opposite sides of the box in T2, whereas, in T3, the animals explored the same objects with one of the objects remaining in its location, and the other object moved to a new location in the open field (Figure 2 (a)). The movement of the mouse in T2 and T3 was video‐tracked. Young adult mice displayed competence for object location memory, which was evident from their preference to explore the object in a novel place (OINP) over the object in a familiar place (OIFP) (*p* < 0.001; Figure S1 (h)). However, 18‐month‐old mice showed object location memory dysfunction, as they explored OINP and OIFP for similar periods (*p* > 0.05; Figure S1 (i)). The TOETs were higher in young adults than 18‐month‐old mice (*p* < 0.01; Figure S1 (j–k)) in T2–T3, which further confirmed the reduced motor activity in late middle age.

The OLT in untreated aged mice demonstrated the permanence of object location memory dysfunction. Representative tracings depicting the movement and exploration of objects by an aged mouse and an aged‐MET mouse in T2‐T3 are illustrated in Figure 2 (b1–b2). Impaired memory in aged mice was evident from their exploration of the OIFP for longer durations than the OINP (*p* < 0.01) (Figure 2 (c1)). In contrast, aged‐MET mice displayed improved recognition memory function by their increased preference to explore the OINP over the OIFP in T3 (*p* < 0.05, Figure 2 (c2)). The TOETs in T2 and T3 were comparable between the two groups (*p* > 0.05, Figure 2 (d1‐d2)). Thus, hippocampus‐dependent location memory function could be improved in aged mice through long‐term MET treatment.

### 3.3 MET treatment in late middle age improved recognition memory dysfunction

Animals were next probed with a novel object recognition test (NORT), a behavioral test for recognition memory that examines the ability of animals to distinguish a new object from a familiar object. The first two trials involved the exploration of an empty open field apparatus (T1) and the exploration of two identical objects placed on opposite sides of the open field apparatus (T2) (Figure 2 (e)). The T3 involved placing the mouse in the middle of the open field apparatus with one of the objects from T2 remaining in the same location (familiar object, FO) but the second object replaced with a novel object (NO) (Figure 2 (e)). The function of the perirhinal cortex and the hippocampus is crucial for maintaining recognition memory function (Langston & Wood, 2010). In NORT, the display of intact recognition memory in animals becomes apparent when they preferentially explore the NO instead of the FO in T3. Young adult mice showed a proficiency for novel object recognition, which was apparent by their predilection to explore the NO over the familiar object FO (*p* < 0.01; Figure S2 (b)). On the other hand, 18‐month‐old mice exhibited impaired recognition memory, as they explored FO and NO for comparable durations (*p* > 0.05; Figure S2 (c)). The TOET was higher in young adults than 18‐month‐old mice in T2 (*p* < 0.01) but not in T3 (Figure S2 (d–e)).

Untreated aged mice also displayed object location memory dysfunction. Representative tracings depicting the movement and exploration of objects by an aged mouse and an aged‐MET mouse in T2 and T3 are illustrated in Figure 2 (F1–F2). Aged mice displayed impaired recognition memory, which was evident from their lack of preference to explore the NO in T3 (*p* < 0.05, Figure 2 (G1)). In contrast, recognition memory ability in aged‐MET mice was conspicuous by their preference to explore the NO over the FO in T3 (*p* < 0.001, Figure 2 (g2)). The TOETs in T2 and T3 were comparable between the two groups (*p* > 0.05, Figure 2 (h1–h2)). Thus, better recognition memory function could be achieved in old age by long‐term MET treatment commencing in late middle age.

### 3.4 Ten weeks of MET treatment enhanced the expression of p‐AMPK in neurons

**Figure 3:**
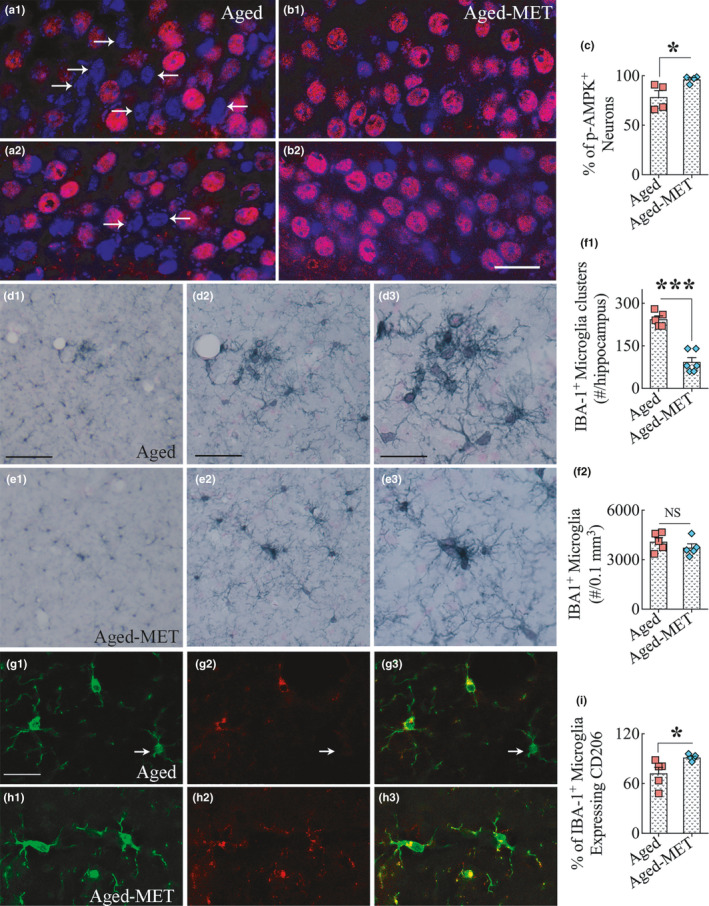
Ten weeks of MET treatment to 18‐month‐old mice enhanced the expression of phosphorylated adenosine monophosphate‐activated protein kinase (p‐AMPK) in neurons. a1‐b2 are examples showing the expression of p‐AMPK in the hippocampal CA3 pyramidal neurons from aged mice (a1, a2) and aged‐MET mice (b1, b2). The sections were processed for p‐AMPK immunofluorescence, followed by DAPI staining. Note that several neurons displaying larger DAPI+nucleus in aged mice lack p‐AMPK expression (arrows in a1, a2). In contrast, virtually all neurons displaying larger heterochromatic DAPI+nucleus in aged‐MET mice have robust p‐AMPK expression. The bar chart C compares the percentage of neurons that express p‐AMPK in the hippocampal CA3 pyramidal cell layer between the aged and aged‐MET groups. *p < 0.05. Scale bar, a1‐b2 = 25 μm. d1–e3 illustrates the distribution of IBA‐1+ microglial clusters in the hippocampus of an aged mouse (d1–d3) and an aged‐MET mouse (e1–e3). Note that microglial clusters are much larger in the aged mice in comparison to smaller clusters in the aged‐MET mice. The bar chart f1 shows that aged‐MET mice displayed a significantly reduced number of microglial clusters than untreated aged mice. The bar chart f2 shows that MET treatment did not alter microglial density in the aged mice. ***p < 0.001, NS, not significant. Scale bar, d1, e1 = 100 μm, d2, e2 = 50 μm, d3, e3 = 25 μm. The figures g1‐h3 illustrate the distribution of IBA‐1+ microglia (green) expressing CD206 (red, representing an M2 phenotype) in the hippocampus of an untreated aged mouse (g1‐g3) and an aged‐MET mouse (h1‐h3). Arrows indicate IBA‐1+ positive microglia lacking CD206 expression (i.e., inflammatory M1 type microglia). The bar chart in I shows that aged‐MET mice displayed a significantly higher number of M2 microglial phenotypes than untreated aged mice. *p < 0.05. Scale bar, g1‐h3 = 25 μm

We first ascertained whether 10 weeks of MET was sufficient to enhance the expression of p‐AMPK in neurons belonging to the cerebral cortex and the hippocampus. A majority of neurons in both aged mice and aged‐MET mice expressed p‐AMPK in these regions. However, the expression was patchy in aged mice receiving no treatment, with a sizeable fraction of neurons displaying either a low intensity of expression or completely lacking the expression of p‐AMPK (Figure 3 (a1–a2)). In contrast, virtually all neurons in aged‐MET mice displayed mostly a uniform expression of p‐AMPK (Figure 3 (b1–b2)). Quantification in the CA3 pyramidal cell layer revealed that a significantly higher percentage of neurons expressed p‐AMPK in aged‐MET mice than aged mice receiving no treatment (*p* < 0.05, Figure 3 (c)). Thus, 10 weeks of MET treatment leads to enhanced expression of p‐AMPK in neurons of aged mice.

### 3.5 MET treatment reduced microglial clustering and preserved a larger population of M2 phenotype

We processed serial brain tissue sections for ionized calcium‐binding adaptor molecule 1 (IBA‐1) immunostaining. We examined whether 10 weeks of MET treatment in aged mice altered the distribution or morphology of microglia, one of the principal mediators of chronic inflammation in the aged brain. We first examined the occurrence of clustering of microglia, a pathological feature consistently seen in the aged hippocampus (Figure 3 (d1–d3)). Such microglial clusters reflect ongoing neurodegenerative processes, including dendritic regression, loss of synapses, and axonal degeneration in the aged hippocampus. In aged‐MET mice, the overall occurrence of such clusters was reduced, and when present, the clusters were much smaller in size and contained fewer microglia (Figure 3 (e1–e3)). Quantification of microglial clusters through the septotemporal axis of the entire hippocampus revealed that MET treatment significantly reduced the number of microglial clusters in the aged hippocampus (*p* < 0.001, Figure 3 (f1)). Stereological counts of IBA‐1+ cells in the hippocampus demonstrated a comparable number of microglia per unit volume between aged and aged‐MET groups (*p* > 0.05, Figure 3 (f2)), suggesting that MET treatment did not alter the overall number of microglia in the hippocampus but decreased the propensity to form clusters. We next examined the fractions of IBA‐1+ microglia that expressed CD206, a marker of M2 microglia, or non‐inflammatory microglia through dual immunofluorescence staining for IBA‐1 and CD206 (Figure 3 (g1–h3)). Quantification revealed that a significantly higher percentage of microglia expressed CD206 in aged‐MET mice than aged mice receiving no treatment (*p* < 0.05, Figure 3 (i)).

**Figure 4:**
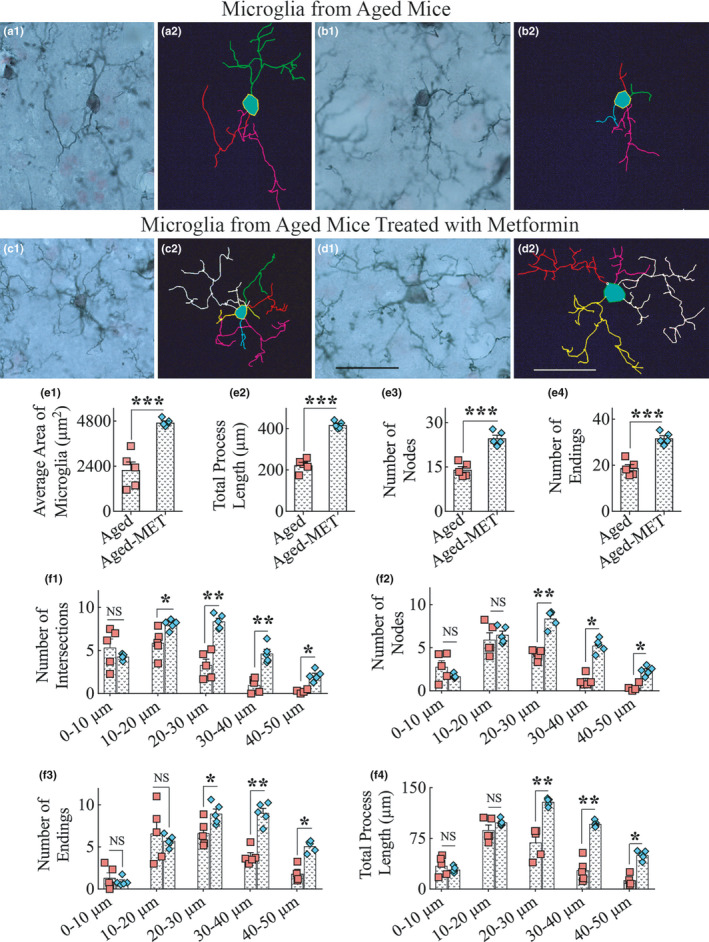
Ten weeks of MET treatment to 18‐month‐old mice resulted in a greater frequency of microglia with highly ramified processes in the hippocampus. a1‐d2 show representative examples of microglial morphology traced with Neurolucida from the hippocampus of aged mice (a1–b2) and aged‐MET mice (c1–d2). Note that microglial cells from untreated aged mice display shorter processes and diminished ramification of processes (a1–b2), whereas microglia from aged‐MET mice show longer and highly ramified processes (c1–d2). The bar charts e1–e4 compare the various morphometric measures between aged mice and aged‐MET mice, which include the average area occupied by individual microglia (e1), the total process length (e2), the number of nodes (e3), and the number of process endings (e4). The bar charts f1–f4 compare the number of intersections (f1), number of nodes (f2), number of process endings (f3), and total process length (f4) between aged mice and aged‐MET group at 0–10 μm, 10–20 μm, 20–30 μm, 30–40 μm, and 40–50 μm distances from the soma. Note that a vast majority of parameters denoting the ramification of processes are enhanced in aged‐MET mice, compared to untreated aged mice. *p < 0.05; **p < 0.01; ***p < 0.001; NS, not significant. Scale bar, a1‐d2 = 25 μm

Thus, 10 weeks of MET treatment to aged mice resulted in significantly reduced microglia clustering and seemed to maintain a higher percentage of non‐inflammatory CD206+ M2 microglia. To further confirm this possibility, we examined whether MET treatment modulated the morphology of microglia. Morphologically, most IBA‐1+ microglia in aged mice appeared activated, resembling an M1 phenotype with visible reductions in the number of processes as well as in the ramification of processes (Figure 4 (a1–b2)). In contrast, a vast majority of microglia in aged‐MET mice resembled an M2 phenotype, a type of microglia exhibiting highly ramified processes occupying a larger area of brain tissue (Figure 4 (c1–d2)). Using Neurolucida and the IBA‐1 immunostained brain tissue sections blinded for experimental groups, we traced microglial processes from individual microglia in a region of the hippocampal CA3 subfield from both aged mice and aged‐MET mice (9–10 microglial cells/animal, *n* = 5 animals/group). Morphometric analyses of traced microglia suggested that MET treatment in late middle age modulated microglia into an M2 phenotype in the aged hippocampus. The M2 features of microglia in the MET‐treated group include the increased area occupied by individual microglia, increased total process length, and a higher number of nodes and endings (*p* < 0.001, Figure 4 (e1–e4)). Furthermore, the Sholl analysis of microglial processes revealed that microglia in the aged‐MET group exhibited a higher number of intersections, nodes, endings, and higher total process lengths at multiple distances from the soma (*p* < 0.05–0.01, Figure 4 (f1–f4)). Thus, MET treatment commencing in late middle age and continuing for 10 weeks maintained a larger population of healthy M2 type of microglia in the aged hippocampus.

### 3.6 MET treatment in late middle age appeared to reduce the occurrence of reactive astrocytes

**Figure 5:**
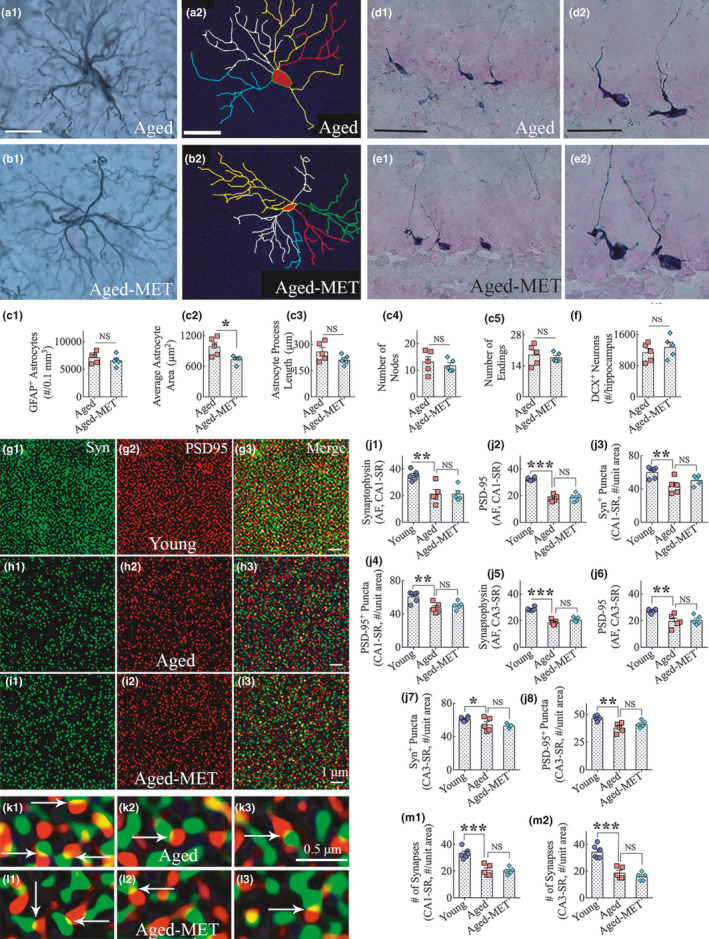
Ten weeks of MET treatment to 18‐month‐old mice reduced astrocyte hypertrophy but had no effects on neurogenesis or synapses in the hippocampus. a1‐b2 show representative examples of astrocyte morphology traced with Neurolucida from the hippocampus of an aged mouse (a1–a2) and an aged‐MET mouse (b1–b2). The astrocyte from an untreated aged mouse displays hypertrophied soma and thicker processes (a1), whereas the astrocyte from an aged‐MET mouse exhibits smaller soma and thinner processes (b1). The bar chart in c1 shows that the density of astrocytes in the hippocampus did not differ between the aged and aged‐MET groups. The bar charts in c2‐c5 compare the area occupied by individual astrocytes (c2), the total process length of individual astrocytes (c3), and nodes (c4) and endings (c5) in astrocyte processes between aged and aged‐MET groups. Note that the area occupied by individual astrocytes (c2) is significantly reduced in the aged‐MET mice, compared to untreated aged mice. *p < 0.05, NS, not significant. Scale bar, a1–b2 = 25 μm. d1–e2 show representative examples of the distribution of doublecortin‐positive (DCX+) newly born neurons in the subgranular zone and granule cell layer (SGZ‐GCL) of the hippocampus from an untreated aged mouse (d1) and an aged‐MET mouse (e1). d2 and e2 are magnified views of regions from d1 and e1. The bar chart F compares the number of DCX+neurons in the SGZ‐GCL between untreated aged and aged‐MET groups. Note that the extent of neurogenesis is comparable between the two groups. NS, not significant. Scale bar, d1, e1 = 50 μm; d2, e2 = 25 μm. g1‐i3** **shows representative images of synaptophysin+ (Syn+) and postsynaptic density 95+ (PSD95+) puncta in the CA3 stratum radiatum of mice belonging to young (g1–g3), untreated aged (h1–h3), and aged‐MET (i1–i3) groups. The bar charts in j1‐j8 compare area fraction (AF) of Syn+ and PSD95+ puncta, and the density of Syn+and PSD95+ puncta per unit area, in the stratum radiatum of CA1 (j1–j4) and CA3 (j5–j8) subfields across young, untreated aged, and aged‐MET groups. k1–l3 illustrates representative images of putative synapses in the CA3 stratum radiatum of mice belonging to untreated aged (k1–k3) and aged‐MET (l1–l3) groups. The bar charts in m1 and m2 compare the density of putative synapses (i.e., junctions of Syn+and PSD95+ puncta) per unit area in the stratum radiatum of CA1 (m1) and CA3 (m2) subfields across young, untreated aged, and aged‐MET groups. Note that Syn+and PSD95+ puncta (j1‐j8) and the number of putative synapses (m1–m2) are reduced in the aged mice compared to the young adult mice but similar between mice belonging to aged and aged‐MET groups. *p < 0.05; **p < 0.01; ***p < 0.001; NS, not significant. Scale bar, g1–I3 = 1 μm; k1–l3 = 0.5 μm

We investigated whether MET treatment modulated the morphology of astrocytes into less reactive phenotypes. Morphologically, most glial fibrillary acidic protein (GFAP) positive astrocytes in aged mice appeared reactive with thick primary and secondary processes, in comparison to mostly thinner processes in aged‐MET mice (Figure 5 (a1, b1)). Using Neurolucida and the GFAP immunostained brain tissue sections blinded for experimental groups, we traced astrocyte processes from individual astrocytes from a region of the hippocampal CA1 subfield from both untreated aged mice and aged mice that received MET (9–10 astrocytes/animal, *n* = 5 animals/group) (Figure 5 (a2, b2)). The number of astrocytes per unit volume of the hippocampus did not differ between the aged and aged‐MET groups (*p* > 0.05, Figure 5 (C1)). However, morphometric analyses revealed that, in comparison to astrocytes from aged mice, astrocytes in the aged‐MET group occupied a reduced area of brain tissue (*p* < 0.05, Figure 5 (c2)) and displayed reduced total process length (~20% reduction, *p* > 0.05, Figure 5 (c3)). The number of nodes and endings in astrocyte processes did not vary between untreated aged mice and aged mice receiving MET (*p* > 0.05, Figure 5 (c4 and c5)), implying that MET treatment reduced the thickening and lengthening of existing processes. Thus, MET treatment commencing in late middle age and continuing for 10 weeks seemed to reduce the occurrence of reactive astrocytes in the aged hippocampus.

### 3.7 MET treatment in late middle age did not increase neurogenesis in the aged hippocampus

We studied whether MET treatment in late middle age altered the extent of hippocampal neurogenesis in old age. We examined the distribution and density of newly born neurons that expressed doublecortin (DCX) in the dentate subgranular zone and the granule cell layer (SGZ‐GCL, *n* = 5 animals/group). The overall distribution and density of DCX+neurons seemed comparable between the aged mice and aged‐MET mice (Figure 5 (d1–e2)). The quantification of DCX+neurons through the entire septotemporal axis of the hippocampus using serial sections revealed no differences in DCX+newly born neuron numbers between untreated aged mice and aged‐MET mice (Figure 5 (f)). Thus, MET treatment commencing in late middle age and continuing for 10 weeks at 100 mg/kg did not enhance the extent of hippocampal neurogenesis in male C57BL/6 J mice.

### 3.8 MET treatment did not induce neosynaptogenesis in the aged hippocampus

We investigated whether 10 weeks of MET treatment commencing in late middle age resulted in neosynaptogenesis in the aged hippocampus. We employed 0.5‐micrometer thick Z‐sectioning of brain tissue sections from young adult, aged, and aged‐MET groups processed for synaptophysin (Syn) and postsynaptic density protein 95 (PSD95) dual immunofluorescence. Representative images of Syn+ and PSD95+ puncta in the CA3 stratum radiatum of mice belonging to young, aged, and aged‐MET groups are illustrated in Figure 5 (g1‐i3). We first performed area fraction analysis of Syn+ and PSD95+ puncta using ImageJ and then quantified the density of Syn+ and PSD95+ puncta per unit area in the stratum radiatum of CA1 and CA3 subfields. Such analyses demonstrated reduced Syn+ and PSD95+ puncta in the aged mice compared to the young adult mice (*p* < 0.05–0.001, Figure 5 (j1‐j8)). Nonetheless, both area fraction and the density of Syn+ and PSD95+ puncta were similar between mice belonging to aged and aged‐MET groups (*p* > 0.05, Figure 5 (j1‐j8)). We also examined the occurrence of putative synapses (i.e., junctions of Syn+ and PSD95+ puncta) in the stratum radiatum of CA1 and CA3 subfields. Representative images of putative synapses in the CA3 stratum radiatum of mice belonging to aged and aged‐MET groups are shown in Figure 5 (k1‐l3). Measurement of the density of putative synapses in the stratum radiatum of CA1 and CA3 demonstrated a similar trend with aged mice showing a decrease compared to the young adult mice (*p* < 0.001, Figure 5 (m1, m2)), and aged and aged‐MET mice showing no differences between them (*p* > 0.05, Figure 5 (m1‐m2)). Thus, MET treatment did not induce neosynaptogenesis in the hippocampus of male C57BL/6 J mice.

### 3.9 MET treatment in late middle age reduced neuroinflammatory markers in the hippocampus

**Figure 6:**
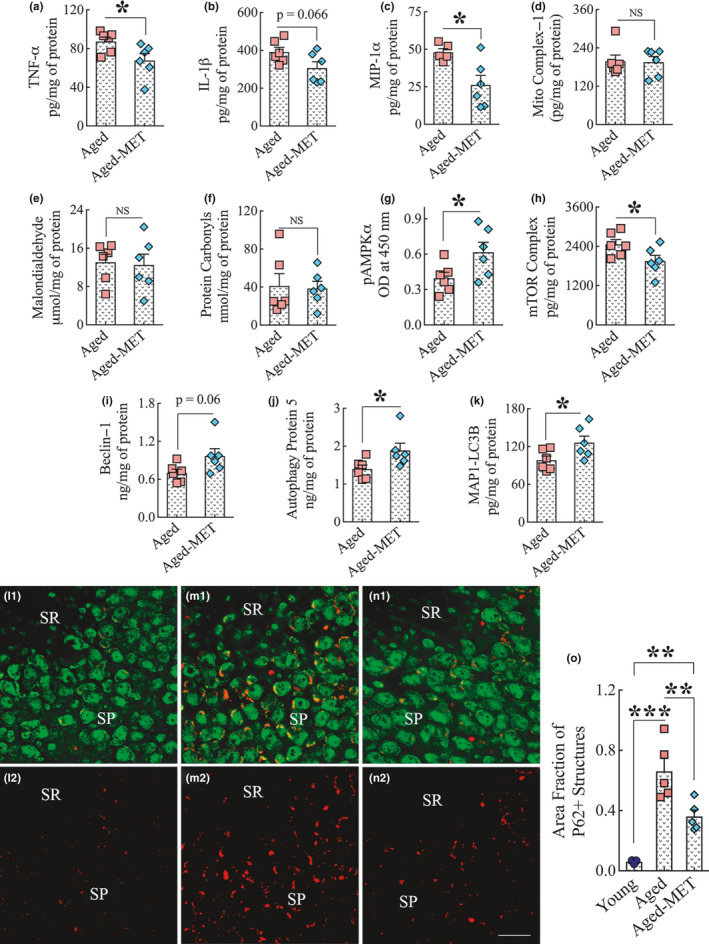
Two weeks of MET treatment to 18‐month‐old mice reduced proinflammatory markers, activated AMPK, inhibited the mammalian target of rapamycin (mTOR), and enhanced autophagy in the hippocampus without altering the concentration of mitochondrial complex 1, or the oxidative stress markers malondialdehyde and protein carbonyls. The bar charts a‐c show a reduced concentration of proinflammatory markers TNF‐α (a), IL‐1β (b), and MIP‐1α (c) in the hippocampus of the MET‐treated aged mice compared to untreated aged mice. The bar charts (d–f) show that the concentration of mitochondrial complex I (d), malondialdehyde (e), and protein carbonyls (f) in the hippocampus did not differ between untreated aged mice and MET‐treated aged mice. The bar charts in g–h show that MET treatment increased the concentration of pAMPKα (g) and decreased the level of mTOR complex (h) in the hippocampus. The bar charts i‐k show MET treatment enhanced the levels of autophagy‐related proteins beclin‐1 (i), autophagy protein 5 (j), and microtubule‐associated protein 1 light chain 3 beta (MAP1‐LC3B; k) in the hippocampus. *p < 0.05. l1‐n2 shows representative images of p62 and NeuN dual immunofluorescence in the CA3 subfield of the young mouse (l1–l2), untreated aged mouse (m1–m2), and the aged mouse that received 10 weeks of MET treatment commencing in late middle age (n1, n2) mice. The bar chart (o) compares the area fraction of p62+ structures in the CA3 subfield of the hippocampus across three groups. Note that the area fraction of p62+ structures is decreased in MET‐treated aged mice compared to the untreated aged mice. SP, stratum pyramidale; SR, stratum radiatum; NS, not significant. **p < 0.01; ***p < 0.001. Scale bar, l1‐n2 = 25 μm

To understand the molecular mechanisms underlying MET‐mediated beneficial effects on the aged hippocampus, we measured the concentration of several neuroinflammatory and oxidative stress markers in the hippocampus of 18‐month‐old mice receiving MET (100 mg/kg/day, once daily) or no treatment for 2 weeks (*n* = 6/group). In comparison to untreated mice, MET‐treated mice displayed reduced concentration of tumor necrosis factor‐alpha (TNF‐α, *p* < 0.05, Figure 6 (a)), interleukin‐1 beta (IL‐1β, *p* = 0.066, Figure 6 (b)), and macrophage inflammatory protein‐1 alpha (MIP‐1α, *p* < 0.05, Figure 6 (c)), implying that MET treatment in late middle age leads to reduced inflammation in the hippocampus. However, MET treatment in late middle age did not alter the levels of mitochondrial complex 1, or the oxidative stress markers malondialdehyde (MDA) and protein carbonyls in the hippocampus (*p* > 0.05, Figure 6 (d–f)), suggesting that the dose of MET employed in the study did not interfere with the activity of mitochondrial complex I or the extent of oxidative stress in the hippocampus of 18‐month‐old mice.

### 3.10 MET treatment in late middle age enhanced pAMPK and inhibited mTOR in the hippocampus

To further comprehend the mechanisms of MET‐mediated positive effects on the aged hippocampus, we examined the concentration of phosphorylated AMPK alpha (pAMPKα) and mTOR complex (mTORC1+mTORC2) in the hippocampus of 18‐month‐old mice receiving MET (100 mg/kg/day, once daily) or no treatment for 2 weeks (*n* = 6/group). In comparison to untreated mice, MET‐treated mice displayed increased concentration of pAMPKα (*p* < 0.05, Figure 6 (g)) and reduced concentration of mTOR complex (*p* < 0.05, Figure 6 (h)) in the hippocampus. The results suggested that two weeks of MET treatment in late middle age is sufficient for reducing mTOR signaling in the hippocampus through activation of AMPK, which is consistent with a previous study showing that MET treatment suppresses mTOR signaling through activated AMPK in other organs (Miller et al., 2013).

### 3.11 MET treatment in late middle age enhanced autophagy in the hippocampus

Aging and neurodegenerative disorders are associated with diminished autophagy in the brain (Glatigny et al., 2019; Scrivo et al., 2018), increased mTOR signaling inhibits autophagy (Bockaert & Marin, 2015), and MET treatment leads to increased autophagy through inhibition of mTOR in other systems (Bharath et al., 2020). Therefore, we examined the concentration of several autophagy markers in the hippocampus of 18‐month‐old mice receiving MET (100 mg/kg/day, once daily) or no treatment for 2 weeks (*n* = 6/group). The autophagy markers included beclin‐1, autophagy protein 5 (ATG5), and microtubule‐associated protein 1 light chain 3 beta (MAP1‐LC3B). In comparison to untreated mice, MET‐treated mice displayed increased concentration of beclin‐1 (*p* = 0.06, Figure 6 (i)), ATG5 (*p* < 0.05, Figure 6 (j)), and MAP1‐LC3B (*p* < 0.05, Figure 6 (k)) in the hippocampus. The results suggested that 2 weeks of MET treatment in late middle age is sufficient for enhancing autophagy in the hippocampus, mediated likely through the activation of AMPK and suppression of mTOR signaling.

We also examined whether 10 weeks of MET treatment commencing in late middle age maintained enhanced autophagy in the aged hippocampus. We employed 2.0‐micrometer thick Z‐sectioning of brain tissue sections from young adult, aged, and aged‐MET groups processed for p62 and neuron‐specific nuclear protein (NeuN) dual immunofluorescence (*n* = 5/group). The expression of p62 in cells is used as a reporter of autophagic activity because p62 delivers ubiquitinated cargoes for autophagic degradation and acts as a substrate during autophagic degradation (Myeku et al., 2011). Since the intracellular level of p62 is dependent on post‐translational autophagic degradation, increased accumulation of p62 implies reduced autophagy. Representative images of p62+ structures in the CA3 pyramidal neurons of mice belonging to young, aged, and aged‐MET groups are illustrated in Figure 6 (l1‐n2). The overall density of p62+ structures in CA3 pyramidal neurons appeared higher in the untreated aged mouse (Figure 6 (m2)) compared to both the young mouse (Figure 6 (l2)) and the aged mouse receiving MET treatment (Figure 6 (n2)). Therefore, we performed area fraction analysis using ImageJ, which confirmed increased p62+ structures in the aged mice compared to young adult mice (*p* < 0.001, Figure 6 (o)). Notably, 10 weeks of MET treatment significantly reduced p62+ structures in the hippocampal CA3 pyramidal neurons of aged mice (*p* < 0.01, Figure 6 (o)), implying enhanced autophagy.

### 3.12 MET treatment in late middle age does not significantly alter blood glucose concentration

We measured non‐fasting blood glucose concentration in 18‐month‐old animals that received MET or no treatment for two weeks (*n* = 6/group). In MET‐treated animals, the blood was collected 3 h after the last MET administration. Although blood glucose concentration was reduced in MET‐treated animals by 13%, the reductions were not significant statistically (aged, mean ± SEM = 148.1 ± 7.0 mg/dl; aged‐MET, 129.0 ± 10.4 mg/dl, *p* > 0.05). Thus, MET's beneficial effects on aged brain function do not appear to be mediated through better glucose metabolism.

## 4 DISCUSSION

The study demonstrated that 10 weeks of MET treatment commencing in late middle age leads to better brain function in old age. The beneficial functional changes comprised improved proficiency for pattern separation and location and recognition memories in old age. The positive cellular modifications facilitated by MET treatment in the hippocampus encompassed significant modulation of microglia into a beneficial antiinflammatory M2 phenotype and reduced hypertrophy of astrocytes. MET treatment also induced several favorable molecular changes in the hippocampus, including the reduced concentration of proinflammatory cytokines and enhanced autophagy concomitant with the activation of AMPK and inhibition of mTOR signaling. However, MET treatment did not alter the extent of neurogenesis or induce neosynaptogenesis in the hippocampus, suggesting that increased production of new neurons or sprouting of new synapses in the hippocampus are not among the mechanisms by which MET improved hippocampus function. Furthermore, MET treatment did not significantly reduce the non‐fasting blood glucose concentration in aged mice, which suggested that MET's beneficial effects on the aged brain function are likely not mediated through better glucose metabolism.

The capability of MET to improve cognitive function has been a matter of debate. While some studies in T2DM patients have suggested beneficial effects of MET on cognitive function (Hsu et al., 2011; Ng et al., 2014), other studies implied that long‐term use of MET for T2DM might increase the risk for developing cognitive impairment (Imfeld et al., 2012; Moore et al., 2013). Moreover, in nondiabetic patients with MCI or mild AD‐related dementia, MET treatment did not improve cognitive function (Koenig et al., 2017). The effects of MET in high‐fat diet animal models are also controversial, with three studies showing alleviation of cognitive deficits and another study reporting no improvement (Allard et al., 2016; Lennox et al., 2014; McNeilly et al., 2012; Pintana et al., 2012). In contrast, studies in models where cognitive impairments were induced through drug/chemical treatment, hypoxia, or stress, MET treatment improved cognitive function (Mostafa et al., 2016; Zhao et al., 2019). Thus, the overall effects of MET seemed to vary in different disease conditions.

Intriguingly, a previous study using a mouse model suggested that MET treatment in old age leads to memory impairment (Thangthaeng et al., 2017). The results of the present study sharply contradict these findings, however. The discrepancy between the two studies’ results likely reflects variances in the timing of intervention, the dose, the frequency/duration of administration, and the type of cognitive tests employed. The previous study administered MET for 8 weeks through drinking water at a dose of 2 mg/ml in 22‐month‐old male C57BL6/J mice (Thangthaeng et al., 2017), whereas the current study administered MET at 100 mg/kg via oral gavage for 10 weeks in 18‐month‐old C57BL6/J mice. First, the commencement of MET treatment to 18‐month‐old mice vis‐à‐vis to 22‐month‐old mice could have differential effects as the extent of neuroinflammation and neuronal dysfunction increases progressively with aging in the brain (Castellano et al., 2017; Lana et al., 2016). Also, 18‐month‐old mice denote a late middle age, whereas 22‐month‐old mice represent old age. Therefore, it is plausible that commencement of MET treatment in late middle age can mediate beneficial effects, compared to commencement of MET treatment in old age, a stage at which neuroinflammation and neuronal dysfunction have progressed to much higher levels. Second, while the differences in the duration of treatment (8 weeks versus 10 weeks) seemed somewhat minimal, the mode of MET administration varied significantly between the two studies. The administration of MET throughout the day via drinking water in the previous study (Thangthaeng et al., 2017) versus a bolus administration via oral gavage in the present study could result in distinctive effects. Notably, it is unknown whether MET administration at 2 mg/ml via drinking water throughout the day would result in sufficient concentration of MET in the brain to induce an increased expression of p‐AMPK because MET concentration depends on the drinking behavior of mice over 24 hours. Since enhanced expression of p‐AMPK is one of the primary mechanisms by which MET produces beneficial effects (Rotermund et al., 2018), this issue is important. Indeed, in our study, both 2 weeks and 10 weeks of MET treatment regimen via oral gavage at 100 mg/kg did result in an increased expression of p‐AMPK.

Furthermore, the previous study did not find significant cognitive impairment as learning curves in a water maze test were comparable between MET‐treated and untreated mice. Also, memory impairment in the previous study was solely based on spatial memory retrieval ability differences after water maze learning. In contrast, the current study employed three different behavioral tests that examined several cognitive and memory function modalities. Specifically, the tests in the current study examined the ability of animals for functions dependent on the dentate gyrus and the CA3 subfield (i.e., the ability for pattern separation in a PST), the dorsal hippocampus (i.e., the object location memory in OLT), and both hippocampus and the perirhinal cortex (i.e., the object recognition memory in NORT). In all three cognitive tests, MET‐treated aged animals showed improved cognitive and memory function than untreated aged mice, which comprised enhanced proficiency for pattern separation and location and recognition memories.

A progressive increase in the extent of neuroinflammation in the hippocampus is one of the hallmarks of aging (Filfan et al., 2020; Shetty et al., 2018). Such neuroinflammation is typified by the activation of microglia and hypertrophy of astrocytes and increased concentration of multiple proinflammatory cytokines secreted mostly by activated microglia and reactive astrocytes (Clarke et al., 2018; Lana et al., 2016). Aging is also associated with systemic inflammation, which can also cause proinflammatory changes in the hippocampus and deficits in the long‐term potentiation (a basis of learning and memory) in middle‐aged animals, and antiinflammatory treatment can alleviate such adverse effects (Shetty et al., 2018). In this study, MET treatment mediated antiinflammatory effects, which could be seen from the reduced clustering of microglia, alternative activation of microglia into an M2 phenotype, and reduced astrocyte hypertrophy. Furthermore, two weeks of MET treatment in 18‐month‐old mice resulted in a reduced concentration of proinflammatory cytokines TNF‐α, and IL‐1β, and the chemotactic cytokine MIP‐1α in the hippocampus. These results are consistent with the antiinflammatory effects of MET observed in several previous studies. For example, MET treatment inhibited NF‐kB signaling as well as reduced proinflammatory cytokines in different cell types (Cameron et al., 2016; Isoda et al., 2006). Also, in a mouse model of Parkinson's disease, MET reduced the numbers of microglia and proinflammatory cytokines TNF‐α, IL‐1β, IL‐6, and iNOS (Ismaiel et al., 2016; Lu et al., 2016). MET treatment has also been shown to protect the astrocytes against apoptosis and cell death induced by oxygen and glucose deprivation (Gabryel & Liber, 2018). Moreover, in a stroke model, MET‐induced AMPK activation changed the microglia polarization toward the antiinflammatory M2 phenotype, which aided tissue repair following stroke‐induced injury (Jin et al., 2014). Neuroinflammation can adversely affect cognitive function. For instance, increased concentration of TNF‐α, IL‐1β, and MIP‐1α could impair synaptic transmission and plasticity in the hippocampus, and memory function (Lynch, 1998; Ma et al., 2015; Marciniak et al., 2015). Thus, the glial plasticity and suppression of TNF‐α, IL‐1β, and MIP‐1α levels mediated by MET in the aged hippocampus are likely among the principal mechanisms by which MET treatment improved brain function in old age. These results are congruous with the emerging concept that neuroinflammation is linked to cognitive dysfunction in aging and that the modulation of chronic neuroinflammation in aging is beneficial for improving cognitive function (Carlessi et al., 2019; Navakkode et al., 2018).

We also examined whether MET treatment commencing in late middle age improved the extent of hippocampal neurogenesis or synaptic density in old age. Measurement of the number of newly born neurons and Syn+ and PSD95+ puncta in the hippocampus revealed no differences between untreated aged mice and MET‐treated aged mice, implying that 10 weeks of MET treatment is not efficient for enhancing the extent of hippocampal neurogenesis or inducing neosynaptogenesis in male C57BL/6 J mice. The neurogenesis results are consistent with a recent study showing that MET does not improve subventricular zone neurogenesis in male C57BL/6 J mice (Ruddy et al., 2019). The study also suggested that only female mice respond to MET treatment with enhanced neurogenesis because of hormonal influence on neural stem cell (NSC) proliferation. Specifically, the study suggested that estradiol in females creates a permissive environment for NSCs to proliferate in response to MET, whereas testosterone in males creates an inhibitory environment for NSCs to proliferate in response to MET (Ruddy et al., 2019). Another study showed that organic cation transporters (OCTs) involved in the transportation of MET for urinary secretion are lower in female kidneys due to the effect of sex steroids, which likely leads to higher MET accumulation in other organ systems of females to mediate its beneficial effects (Ma et al., 2016). Thus, higher and more frequent doses of MET are likely needed for improving neurogenesis in males. Nonetheless, the current study results suggest that MET treatment in late middle age could improve cognitive function in male mice without affecting the extent of neurogenesis or enhancing synaptic density in the hippocampus.

MET treatment may have also modulated several other age‐related adverse effects such as neuronal dendritic regression, altered gene expression, and other factors affecting hippocampal plasticity and network activity underlying cognitive, memory function (Burke & Barnes, 2006). MET is capable of modulating the above changes as it acts on central metabolism as well as several major signaling pathways, including AMPK, mTOR, and PI3K‐Akt signaling pathways. MET treatment can also act on the mTOR pathway via AMPK independent routes, which include the inhibition of transcription factors (Nair et al., 2013), and the PI3 K/AKT pathway (Slomovitz & Coleman, 2012) as well as direct inhibition of mTORC1 (Kalender et al., 2010). Indeed, 2 weeks of MET treatment to 18‐month‐old mice in this study increased the expression of pAMPKα and reduced the concentration of the mTOR complex. mTOR signaling integrates upstream signals such as nutrient and redox status and then controls downstream processes such as cellular growth, motility, survival, and death (Laplante & Sabatini, 2012). The mTOR pathway is also crucial for regulating autophagy, a process that is impaired in aging and defective in neurodegenerative diseases (Bockaert & Marin, 2015; Glatigny et al., 2019; Scrivo et al., 2018). Analyses of several proteins implicated in autophagy also uncovered enhanced autophagy in the hippocampus of MET‐treated mice in this study. MET treatment increased the concentration of beclin‐1, a key protein influencing autophagosome formation and maturation (Liang et al., 1999), ATG5, a protein involved in autophagy vesicle formation (Okerlund et al., 2017), and MAP1‐LC3B, a protein having a significant role in phagophore membrane elongation and interaction with p62 (Galluzzi et al., 2017). Furthermore, p62+ structures (a reporter of autophagic activity) were considerably reduced in the hippocampal CA3 pyramidal neurons of aged mice that received 10 weeks of MET treatment, suggesting enhanced autophagy. AMPK activation, inhibition of mTOR signaling, and enhanced autophagy have a role in maintaining normal cognitive function (Glatigny et al., 2019; Kobilo et al., 2014). Therefore, much‐improved brain function observed in MET‐treated aged male mice in this study could be due to a combination of antiinflammatory effects, AMPK activation, mTOR inhibition, and enhanced autophagy in hippocampal neurons. Additional studies are needed in the future to examine whether metformin will have similar results in female mice.

## 5 CONCLUSIONS

Our results in a mouse model of aging provide novel evidence that 10 weeks of MET treatment commencing in late middle age is efficacious for improving cognitive function in the old age. Additional analyses suggested that improved cognitive function following MET treatment is linked to antiinflammatory effects, the activation of AMPK, inhibition of mTOR signaling, and enhanced autophagy in the aged hippocampus. These results imply that MET therapy in middle age leads to better cognitive and memory function in old age.

## CONFLICT OF INTEREST

The authors declare no conflicts of interest.

## AUTHOR CONTRIBUTIONS

Concept: AKS. Research design: MK, SA, LNM, and AKS. Data collection, analysis, and interpretation: MK, SA, LNM, BS, RU, JJG, XR, and AKS. Preparation of figure composites: MK, SA, and AKS. Manuscript writing: MK and AKS. All authors provided feedback and edits to the manuscript text and approved the final version of the manuscript.

## Supporting information

Supplementary Material

## Data Availability

The data that support the findings of this study are available from the corresponding author upon reasonable request.
