# Supplementary material for: Metformin treatment in late middle age improves cognitive function with alleviation of microglial activation and enhancement of autophagy in the hippocampus
Source: Aging Cell. 2021 Jan 14;20(2):e13277. doi: 10.1111/acel.13277 (PMC7884047; doi:10.1111/acel.13277)
Supplement: Supplementary file 1 — Supplementary Material [file ACEL-20-e13277-s001.docx]

**Supplemental File**

**Metformin Treatment in Late Middle Age Improves Cognitive Function with Alleviation of Microglial Activation and Enhancement of Autophagy in the Hippocampus**

**Maheedhar Kodali, Sahithi Attaluri, Leelavathi N Madhu, Bing Shuai, Raghavendra Upadhya, Jenny Jaimes Gonzalez, Xiaolan Rao, and Ashok K. Shetty^§*^**

Institute for Regenerative Medicine, Department of Molecular and Cellular Medicine, Texas A&M University College of Medicine, College Station, Texas, USA

**^§^**Address of Correspondence:

Ashok K. Shetty, PhD

Associate Director and Professor

Institute for Regenerative Medicine

Texas A&M Health Science Center, College of Medicine,

1114 TAMU, 206 Olsen Boulevard

College Station, TX 77843

E-mail: [akskrs@tamu.edu](mailto:akskrs@tamu.edu)

**Supplementary Figure 1**

**
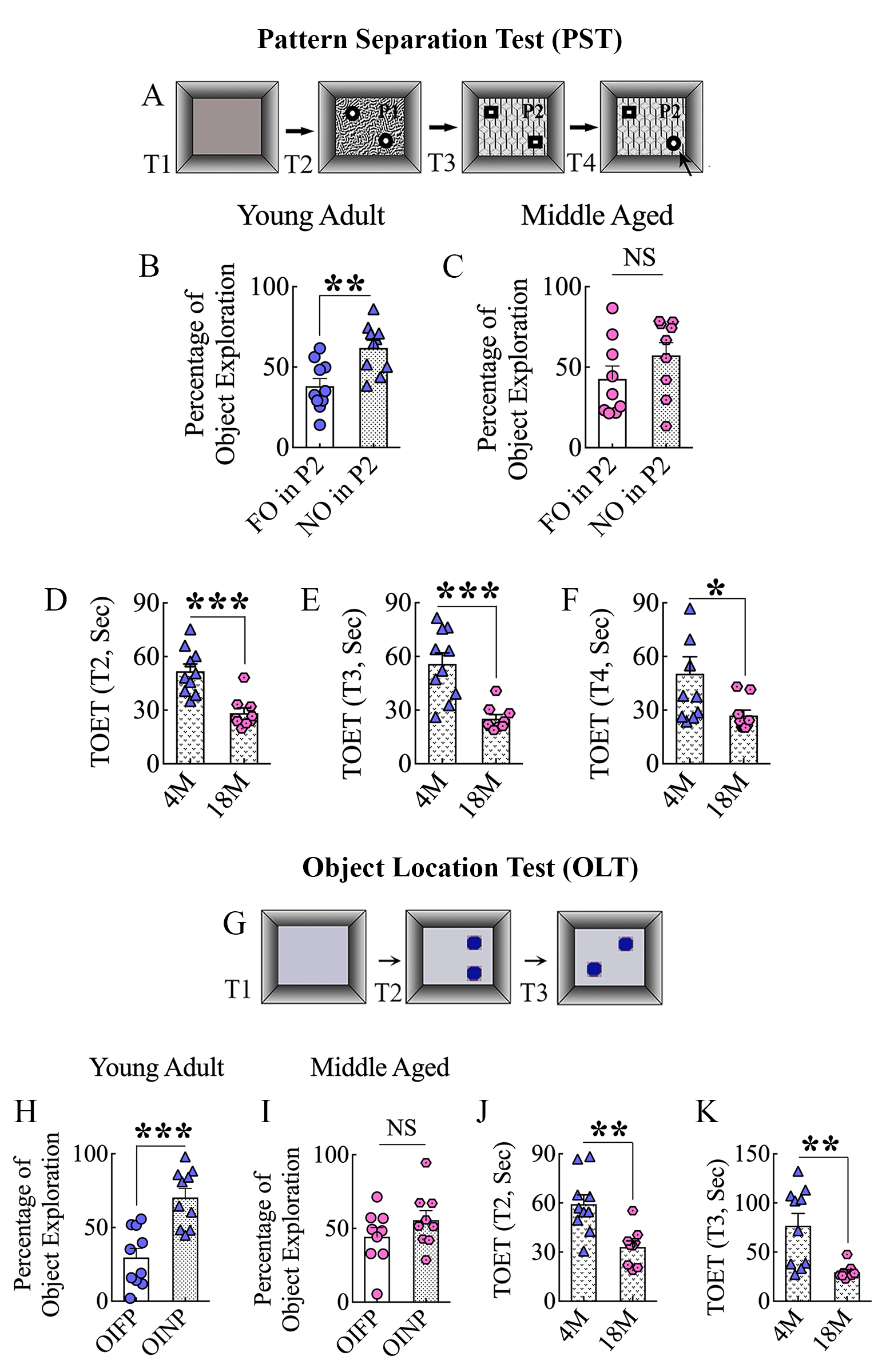
**

The cartoon in A shows the various trials (T1-T4), objects, and floor patterns involved in a pattern separation test. The bar charts in B and C compare the percentage of exploration of familiar and novel objects on pattern 2 (FO or NO on P2) in T4 by young adult mice (B) and middle-aged (18 month old) mice (C). Note that young adult mice displayed a preference for exploring NO on P2 in comparison to middle-aged mice showing no such preference. The bar charts in D-F compare total object exploration times (TOETs) in T2-T4. Cartoon G illustrates the trials (T1-T3) and objects employed in an object location test. The bar charts in H and I compare the percentage of exploration of the object in a familiar place (OIFP) vis-à-vis the object in a novel place (OINP) in T3 by young adult mice (H) and middle-aged mice (I). Note that young adult mice showed a higher tendency for exploring the OINP than the OIFP, in comparison to middle-aged mice showing no such preference. The bar charts in J and K compare TOETs in T2-T3. *, p<0.05; **, p < 0.01; ***, p<0.001 NS, not significant.

**Supplementary Figure 2**

**
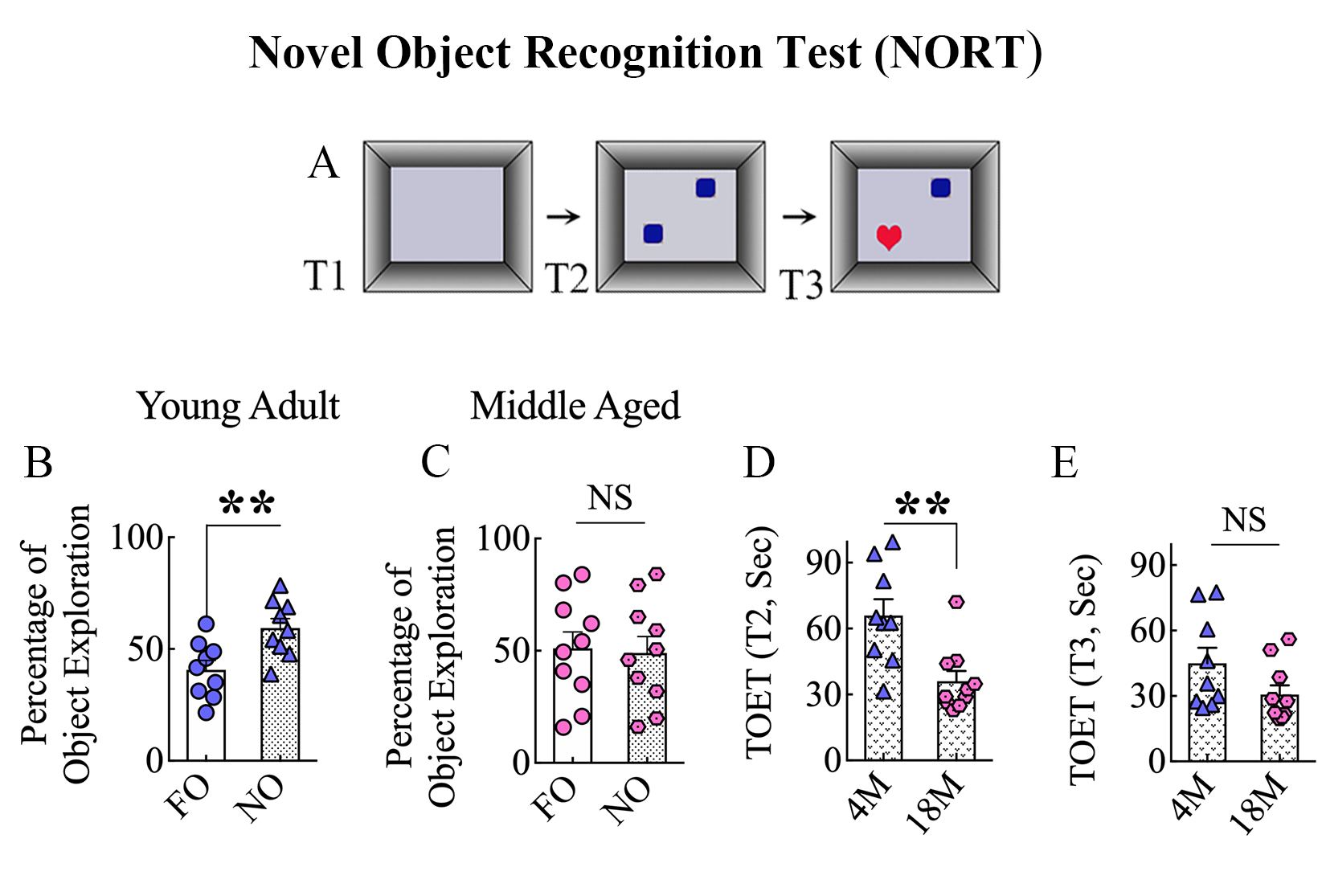
**

The cartoon in A shows the various trials (T1-T3) and objects employed in a novel object recognition test. The bar charts in B and C compare the percentage of exploration of familiar and novel objects (FO or NO) in T3 by young adult mice (B) and middle-aged mice (C). Note that young adult mice preferred to explore the NO over FO (B) whereas, the middle-aged mice showed no such preference (C). The bar charts in D and E compare total object exploration times (TOETs) in T2-T3. **, p < 0.01; NS, not significant.

**Materials and Methods**

***Pattern separation test (PST)***

The PST employed in this study encompassed four consecutive trials (T1-T4), with each lasting 5 minutes and separated by an ITI of 30-minutes. The test was performed as described in our recent reports [Jain et al., 2012; Long et al., 2017; Upadhya et al., 2019a; Shetty et al., 2020]. The AnyMaze video-tracking system recorded the movement of animals in T2-T4. The animals that explored objects for ≥ 20 seconds in T2 and T3 were included for data analysis, as the ability for pattern separation in T4 requires the memory formation for objects and floor patterns in T2 and T3, and such competence needs exploration of different objects and floor patterns for significant periods in T2 and T3. 94% of the total animals included for PST (i.e., 9-12 animals/group) met this criterion, and the results such as the proportion of times spent in exploring NO on P2 vis-à-vis FO on P2, the TOETs, the total distance moved, and the velocities of movement in different trials were computed and compared.

***Object location test (OLT)***

The OLT also included three trials (T1-T3), with each trial lasting 5 minutes and trials separated by an ITI of 15 minutes. [Hattiangady et al., 2014; Long et al., 2017; Upadhya et al., 2019b]. The movement of animals in T2-T3 was recorded using the AnyMaze video-tracking system. The animals that explored objects for ≥ 20 seconds in T2 were included for data analysis. 85% of the total animals included for OLT (i.e., 9-11 animals/group) were adept at reaching this benchmark. The results, such as the percentage of time spent exploring the OIFP versus OINP in T3, the TOETs, the total distance moved, and the velocities of movement in different trials, were calculated and compared.

***Novel object recognition test (NORT)***

The test comprised three trials (T1-T3), with each lasting 5 minutes and separated by an ITI of 15 minutes. The procedure has been detailed in our previous reports [Hattiangady et al., 2014; Long et al., 2017; Upadhya et al., 2019b]. The AnyMaze video-tracking system recorded the movement of animals in T2-T3. The animals that explored objects for ≥ 20 seconds in T2 were included for data analysis, as the ability for identifying the NO in T3 requires the memory for identical objects explored in T2, and such aptitude needs exploration of identical objects for significant periods in T2 [Warburton and Brown, 2015]. 94% of the total animals included for NORT (i.e., 10-12 animals/group) met this criterion, and the data such as the percentage of times spent in exploring FO versus NO in T3, the TOETs, the total distance moved, and the velocity of movement in different trials, were calculated and compared.

***Tissue processing and immunohistochemistry, and quantification of microglia, astrocytes, and DCX+ newly born neurons***

The animals were deeply anesthetized, perfused with 4% paraformaldehyde, the brain tissues were post-fixed, and processed for cryostat sectioning following cryoprotection, as described in our previous studies [Shetty and Turner, 1999; Rao et al., 2006, 2008]. Thirty-micrometer thick coronal sections through the forebrain were collected serially, and every 15^th^ section through the entire septo-temporal axis of the hippocampus was processed for immunohistochemistry, as described in our previous reports [Hattiangady et al., 2011; Hattiangady and Shetty, 2012; Kodali et al., 2015, 2018]. The primary antibodies comprised of a goat anti-IBA-1 (1:1000, Abcam, Cambridge, MA), a rabbit anti-GFAP (1:2000, DAKO, Santa Carla, CA), and a goat anti-DCX (1:300, Abcam). The secondary antibodies comprised biotinylated anti-rabbit or anti-goat IgG’s (Vector Labs, Burlingame, CA). The peroxidase reaction was developed using diaminobenzidine (Vector Labs) or vector SG (Vector Labs) as chromogens. After a thorough wash, the sections were mounted on gelatin-coated slides, dehydrated, cleared, and coverslipped with permount. We quantified the total number of microglial clusters for the hippocampus by counting the number of clusters in serial sections (every 15^th^) through the entire hippocampus and extrapolating the average number per section with the total number of sections through the hippocampus (n=5-6/group). We employed stereology for measuring the total number of DCX+ neurons in the hippocampus (n=5/group). Serial sections (every 15th) through the entire hippocampus stained for DCX were used to quantify absolute numbers of DCX+ neurons, using a StereoInvestigator system (Microbrightfield) comprising a color digital video camera interfaced with a Nikon E600 microscope. The densities of IBA-1+ microglia and GFAP+ astrocytes per unit volume were also quantified stereologically using three serial sections (every 15th) through mid-region of the hippocampus. The stereological method employed has been described in our earlier reports [Hattiangady et al., 2008; Kodali et al., 2016].

### *Morphometric analysis of IBA-1+ microglia and GFAP+ astrocytes*

### The overall morphology of IBA-1+ microglia and GFAP+ astrocytes from aged and aged-MET groups were measured by tracing the soma and processes using a semi-automatic cell tracing system (Neurolucida, Microbrightfield) linked to a Nikon microscope. A region in the CA3 stratum radiatum of the hippocampus and a region in the CA1 stratum radiatum adjacent to the stratum lacunosum were chosen for quantification of microglia and astrocytes respectively. Both IBA-1+ microglia and GFAP+ astrocytes were chosen randomly in these regions, and investigators who performed tracings were blinded to the experimental groups. Furthermore, to verify constant sampling in all animals, microglia and astrocytes selected for quantification had to meet specific criteria. First, the chosen cell appeared to be stained well, and the soma was located in the middle third of the section’s thickness. Second, no processes appeared to be severed near its soma, and other cells’ processes did not overly cover the processes. In each group, 40 microglia and 50 astrocytes (8-10 cells/animal, 5 animals/group) were individually traced in their entirety using an oil immersion 100X lens. The data, such as the average area occupied by individual cells, the average total process length, and the number of nodes and endings, were computed. To measure the pattern and extent of processes at different distances from the soma in both microglia and astrocytes, Sholl’s concentric circle analysis was performed using the NeuroExplorer component of the Neurolucida program.

***Immunofluorescence staining and confocal microscopic analyses***

For visualization of each marker examined in the study, representative sections (n=3-4/animal) were washed in PBS, treated with the normal donkey serum, and incubated overnight in the primary antibody. For p-AMPK immunofluorescence, sections were treated with anti-rabbit p-AMPK (1:100, Abcam, Cambridge, MA) and donkey anti-rabbit IgG tagged Alexa Flour 594 (1:200, Invitrogen, Grand Island, NY). The sections were then rinsed in PBS, incubated in DAPI solution (1:10,000, Millipore, Burlington, MA), washed thoroughly in PBS, and coverslipped with a slow fade/antifade mounting medium (Invitrogen). For CD206 and IBA-1 dual immunofluorescence, we used a cocktail comprising anti-mouse CD-206 (1:500, Santa Cruz, Dallas, TX) and anti-goat IBA-1 (1:1000, Abcam). The sections next treated with a mixture of donkey anti-mouse IgG tagged with Alexa Flour 594 (1:200, Invitrogen, Grand Island, NY) and donkey anti-goat IgG tagged with Alexa Fluor 488 (1:200, Invitrogen), rinsed in PBS, and coverslipped with a slow fade/antifade mounting medium (Invitrogen). For Syn+ and PSD95+ dual immunofluorescence studies, sections were incubated in a cocktail of antibodies comprising anti-rabbit Syn (1:500, synaptic systems, Goettingen, Germany) and anti-goat PSD95 (1:500, Abcam). The sections were next treated with a mixture of donkey anti-rabbit IgG tagged Alexa Fluor 488 (1:200, Invitrogen) and donkey anti-goat IgG tagged Alexa Fluor 594 (1:200, Invitrogen). For NeuN and p62 dual immunofluorescence, the sections were treated with a cocktail of anti-chicken NeuN (1:2000, Millipore) and anti-guinea pig p62 (1:1000, Progen, Heidelberg, Germany). The sections were next treated with a mixture of donkey anti-chicken IgG tagged Alexa Fluor 488 (1:200, Invitrogen) and donkey anti-guinea pig Alexa Fluor 594 (1:200, Jackson ImmunoResearch, West Grove, Pennsylvania), rinsed in PBS and coverslipped with a slow fade/antifade mounting medium (Invitrogen). The p-AMPK expression among DAPI+ neurons (displaying larger nuclei) and CD206 expression among IBA-1+ microglia were identified using a confocal microscope. The percentages of DAPI+ neurons expressing p-AMPK and IBA-1+ microglia exhibiting CD206 expression were next quantified using two-micrometer thick Z-section images taken from 3 sections/animal (n=4-5/group) in a confocal microscope [Hattiangady and Shetty, 2008; Shetty et al., 2020].

### *Biochemical assays using hippocampal lysates and the serum*

After the dissection of the hippocampus from the harvested brains, lysates were prepared using methods described elsewhere [Mishra et al., 2015; Shetty et al., 2017, 2020; Madhu et al., 2019; Upadhya et al., 2020]. Each hippocampal tissue sample was weighed and lysed in an ice-cold tissue extraction buffer (Life Technologies, Carlsbad, CA) with protease inhibitors (Sigma-Aldrich Corp. St. Louis, MO), using a sonic dismembrator for 30 seconds. The lysed solution was centrifuged, and the supernatant solution’s aliquots were stored at -80° C until further use. The protein concentration in different samples was measured using a Pierce BCA reagent kit (Thermo Fisher Scientific, Waltham, MA). We measured the concentration of proinflammatory markers TNF-α, IL-1β and MIP-1α, mitochondrial complex I, oxidative stress markers MDA and protein carbonyls, pAMPKα, mTOR complex, and autophagy proteins beclin-1, ATG5, and MAP1-LC3B from aged and aged-MET groups (n=6/group), using commercially available kits. We followed the manufacturer's instructions that came with the kits for specific assays. We purchased kits from Signosis (Santa Clara, CA) for measuring TNF-α (EA2203) and IL-1β (EA2508) and LSBio (Seattle, WA) for MIP-1α (LSF4952). The assay kit for mitochondrial complex I was purchased from MyBioSource (MBS912812, San Diego CA), whereas the kits for MDA (10009055) and protein carbonyls (10005020) were from Cayman Chemical (Ann Arbor, MI). The additional kits comprise: (i) pAMPKα phosphorylated at Thr 172 in alpha subunit from Cell signaling (7959C, Danvers, MA); (ii) mTOR comprising two distinct signaling complexes mTORC1 and mTORC2 from Abcam (ab206311); (iii) beclin 1 from Aviva Systems Biology (OKEH03266, San Diego, CA), ATG 5 (MBS2884012) and MAP1LC3B (MBS1601177) from MyBioSource (San Diego, CA). We also measured the concentration of non-fasting glucose in the serum collected from aged and aged-MET groups using an assay kit from Millipore Sigma (GAGO20, Burlington, MA). In the MET group, the measurement was done on serum samples collected three hours after the last MET treatment.

**References**

Hattiangady, B., Kuruba, R., & Shetty, A. K. (2011). Acute Seizures in Old Age Leads to a Greater Loss of CA1 Pyramidal Neurons, an Increased Propensity for Developing Chronic TLE and a Severe Cognitive Dysfunction. *Aging and Disease*, 2(1), 1-17.

Hattiangady, B., Mishra, V., Kodali, M., Shuai, B., Rao, X., & Shetty, A. K. (2014). Object location and object recognition memory impairments, motivation deficits and depression in a model of Gulf War illness. *Frontiers in Behavioral Neuroscience*, 8, 78. doi:10.3389/fnbeh.2014.00078

Hattiangady, B., Rao, M .S., & Shetty, A. K. (2008). Plasticity of hippocampal stem/progenitor cells to enhance neurogenesis in response to kainate-induced injury is lost by middle age. *Aging Cell,* 7(2):207-24. doi: 10.1111/j.1474-9726.2007.00363.x.

Hattiangady, B., & Shetty, A. K. (2008). Aging does not alter the number or phenotype of putative stem/progenitor cells in the neurogenic region of the hippocampus. *Neurobiology of Aging*, 29(1), 129-147. doi:10.1016/j.neurobiolaging.2006.09.015

Hattiangady, B., & Shetty, A. K. (2012). Neural stem cell grafting counteracts hippocampal injury-mediated impairments in mood, memory, and neurogenesis. *Stem Cells Translation Medicine*, 1(9), 696-708. doi:10.5966/sctm.2012-0050

Jain, S., Yoon, S. Y., Zhu, L., Brodbeck, J., Dai, J., Walker, D., & Huang, Y. (2012). Arf4 determines dentate gyrus-mediated pattern separation by regulating dendritic spine development. *PLoS One*, 7(9), e46340. doi:10.1371/journal.pone.0046340

Kodali, M., Hattiangady, B., Shetty, G. A., Bates, A., Shuai, B., & Shetty, A. K. (2018). Curcumin treatment leads to better cognitive and mood function in a model of Gulf War Illness with enhanced neurogenesis, and alleviation of inflammation and mitochondrial dysfunction in the hippocampus. *Brain Behavior and Immunity*, 69, 499-514. doi:10.1016/j.bbi.2018.01.009

Kodali, M., Megahed, T., Mishra, V., Shuai, B., Hattiangady, B., & Shetty, A. K. (2016). Voluntary running exercise-mediated enhanced neurogenesis does not obliterate retrograde spatial memory. *Journal of Neuroscience*, 36(31), 8112-8122. doi:10.1523/JNEUROSCI.0766-16.2016

Kodali, M., Parihar, V. K., Hattiangady, B., Mishra, V., Shuai, B., & Shetty, A. K. (2015). Resveratrol prevents age-related memory and mood dysfunction with increased hippocampal neurogenesis and microvasculature, and reduced glial activation. *Scientific Reports*, 5, 8075. doi:10.1038/srep08075

Long, Q., Upadhya, D., Hattiangady, B., Kim, D. K., An, S. Y., Shuai, B., . . . Shetty, A. K. (2017). Intranasal MSC-derived A1-exosomes ease inflammation, and prevent abnormal neurogenesis and memory dysfunction after status epilepticus. *Proceedings of the* National Academy *of Sciences of the United States of America*, 114(17), E3536-e3545. doi:10.1073/pnas.1703920114

Madhu, L. N., Attaluri, S., Kodali, M., Shuai, B., Upadhya, R., Gitai, D., Shetty A. K. (2019). Neuroinflammation in Gulf War Illness is linked with HMGB1 and complement activation, which can be discerned from brain-derived extracellular vesicles in the blood. *Brain Behavior and Immunity,* 81, 430-443. doi: 10.1016/j.bbi.2019.06.040

Mishra, V., Shuai, B., Kodali, M., Shetty, G. A., Hattiangady, B., Rao, X., Shetty, A. K. (2015). Resveratrol treatment after status epilepticus restrains neurodegeneration and abnormal neurogenesis with suppression of oxidative stress and inflammation. *Scientific Reports,* 5, 17807. doi: 10.1038/srep17807.

Rao, M. S., Hattiangady, B., & Shetty, A. K. (2006). Fetal hippocampal CA3 cell grafts enriched with FGF-2 and BDNF exhibit robust long-term survival and integration and suppress aberrant mossy fiber sprouting in the injured middle-aged hippocampus. *Neurobiology of Disease*, 21(2), 276-290. doi:10.1016/j.nbd.2005.07.009

Rao, M. S., Hattiangady, B., & Shetty, A. K. (2008). Status epilepticus during old age is not associated with enhanced hippocampal neurogenesis. *Hippocampus*, 18(9), 931-944. doi:10.1002/hipo.20449

Shetty, A. K., & Turner, D. A. (1999). Aging impairs axonal sprouting response of dentate granule cells following target loss and partial deafferentation. *Journal of Comparative Neurology*, 414(2), 238-254. doi:10.1002/(sici)1096-9861(19991115)414:2<238::aid-cne7>3.0.co;2-a

Shetty, G. A., Hattiangady, B., Upadhya, D., Bates, A., Attaluri, S., Shuai, B. … Shetty, AK. (2017). Chronic Oxidative Stress, Mitochondrial Dysfunction, Nrf2 Activation and Inflammation in the Hippocampus Accompany Heightened Systemic Inflammation and Oxidative Stress in an Animal Model of Gulf War Illness. *Frontiers in Molecular Neuroscience,* 10, 182. doi: 10.3389/fnmol.2017.00182

Shetty, A. K., Attaluri, S., Kodali, M., Shuai, B., Shetty, G. A., Upadhya, D., . . . Rao, X. (2020). Monosodium luminol reinstates redox homeostasis, improves cognition, mood and neurogenesis, and alleviates neuro- and systemic inflammation in a model of Gulf War Illness. *Redox Biology*, 28, 101389. doi:10.1016/j.redox.2019.101389

Upadhya, D., Hattiangady, B., Castro, O. W., Shuai, B., Kodali, M., Attaluri, S., . . . Shetty, A. K. (2019a). Human induced pluripotent stem cell-derived MGE cell grafting after status epilepticus attenuates chronic epilepsy and comorbidities via synaptic integration. *Proceedings of the* National Academy *of Sciences of the United States of America*, 116(1), 287-296. doi:10.1073/pnas.1814185115

Upadhya, D., Kodali, M., Gitai, D., Castro, O. W., Zanirati, G., Upadhya, R., . . . Shetty, A. K. (2019b). A Model of Chronic Temporal Lobe Epilepsy Presenting Constantly Rhythmic and Robust Spontaneous Seizures, Co-morbidities and Hippocampal Neuropathology. *Aging and Disease*, 10(5), 915-936. doi:10.14336/ad.2019.0720

Upadhya, R., Madhu, L. N,. Attaluri, S., Gitaí, D. L. G., Pinson, M. R., Kodali ,M., … Shetty, A. K. (2020). Extracellular vesicles from human iPSC-derived neural stem cells: miRNA and protein signatures, and anti-inflammatory and neurogenic properties. *Journal of Extracellular Vesicles,* 9(1):1809064. doi: 10.1080/20013078.2020.1809064

Warburton, E. C., & Brown, M. W. (2015). Neural circuitry for rat recognition memory. *Behavioural Brain Research*, 285, 131-139. doi:10.1016/j.bbr.2014.09.050
